# Gunshot-related nerve injuries of the upper extremities: clinical, electromyographic, and ultrasound features in 22 patients

**DOI:** 10.3389/fneur.2023.1333763

**Published:** 2024-01-11

**Authors:** Lisa B. E. Shields, Vasudeva G. Iyer, Yi Ping Zhang, Christopher B. Shields

**Affiliations:** ^1^Norton Neuroscience Institute, Norton Healthcare, Louisville, KY, United States; ^2^Neurodiagnostic Center of Louisville, Louisville, KY, United States; ^3^Department of Neurological Surgery, University of Louisville School of Medicine, Louisville, KY, United States

**Keywords:** neurology, gunshot, upper extremity, electrodiagnostic studies, ultrasound, COVID-19

## Abstract

**Objectives:**

Gunshot wounds of the upper extremities may cause permanent neurovascular injuries, leading to significant morbidity, chronic pain, functional loss, and disability. While there are many reports on the incidence and intraoperative findings in gunshot-related nerve injuries (GSNI) sustained during wars, there is a paucity of details pertaining to GSNI of the upper extremities in civilians. The goal of this paper is to provide the clinical, electrodiagnostic (EDX), and ultrasound (US) findings in 22 patients with GSNI of the upper extremities.

**Methods:**

This is a retrospective study of patients referred for EDX studies to evaluate the presence of nerve injury after sustaining GSWs to the upper extremities. All patients underwent EDX studies, and 16 patients had US evaluations. Numerous metrics were documented including presenting symptoms, neurological abnormalities, EDX findings, and US features.

**Results:**

The forearm was the most frequent location of injury (8 [36%] patients). The ulnar nerve was the most common injured nerve (10 [45%] patients), followed by the brachial plexus (7 [32%] patients). All patients complained of muscle weakness; the most frequently affected muscles were the first dorsal interosseous (FDI) (14 [64%] patients) and abductor pollicis brevis (APB) (11 [50%] patients). Muscle atrophy was noted in 19 (86%) patients, 15 of whom had atrophy of the FDI. Axonotmesis was the type of nerve injury in all patients based on EDX studies. Of the 16 patients who underwent US studies, a neuroma in continuity was noted in 4 (25%) patients and neurotmesis in 1 (6.2%) patient. Eleven (69%) patients had enlarged and/or hypoechoic nerves.

**Conclusions:**

Axonotmesis of the ulnar nerve was the most common finding among patients sustaining gunshot injuries to the upper extremities. EDX and US studies provide valuable insight into the underlying pathophysiology and guidance for management of patients with GSNI of the upper extremities.

## Introduction

According to the Centers for Disease Control and Prevention (CDC), more Americans died of gun-related injuries in 2021 than in any other year on record (1). Gun violence (unintentional, suicide, and homicide) is higher than motor vehicle accidents as the leading cause of trauma-related years of potential life lost (2). The burden of gun violence represents a dire public health issue that primarily affects the younger members of society (3). While gunshot wounds (GSWs) of the upper extremities are not commonly life threatening, they pose a high risk of long-term disability due to neurovascular injuries (4).

The mechanism of peripheral nerve injury caused by firearms involves either a direct transection of the nerve or indirect injury by producing thermal damage, shock waves, laceration secondary to fracture fragment displacement, or compression due to swelling or subacute scar formation (5–9). Traction injuries often spontaneously recover without surgical intervention, however, rupture and avulsion injuries usually require surgery and have a worse prognosis. Patients who undergo surgery within 6 months of the injuries usually have a better outcome (6, 7, 10–12). Additionally, vascular injury and fractures increase the risk of nerve injury after GSWs (9).

Firearm injuries of peripheral nerves can cause neuropraxia, axonotmesis, and/or neurotmesis (Table 1) (12). Low-velocity projectiles often damage nerves by direct impact leading to neuropraxia or axonotmesis, and patients usually have significant return of function within several months. High-velocity injuries are caused by shock waves or cavitation effects with ensuing stretching and compression often outside the path of the projectile and across longer nerve segments. Significant return of function is infrequent with high-velocity projectiles. Unlike the military, most of the gunshot injuries in a community setting inflict low-energy penetrating trauma (9).

**Table 1:** Differentiating between neuropraxia, axonotmesis, and neurotmesis.

**Features**	**Neuropraxia**	**Axonotmesis**	**Neurotmesis**
**EMG findings**	CMAP and SNAP elicitable on stimulating the nerve distal to site of lesion, conduction block on proximal stimulation	Inability to record CMAP and SNAP	Inability to record CMAP and SNAP
**Underlying pathology**	No axonal loss Temporary loss of myelin sheath Impaired impulse conduction across injured nerve segment	Axons damaged leading to Wallerian degeneration Most connective tissues of the endoneurium, perineurium, and epineurium are partially or fully intact	Severe damage to axons, myelin sheath, connective tissue leading to Wallerian degeneration Discontinuity of the nerve Most severe form of nerve injury

CMAP, compound muscle action potentials; SNAP, sensory nerve action potentials.

Clinical examination alone cannot differentiate between neuropraxia, axonotmesis, and neurotmesis (7). EDX can differentiate between neuropraxia and axonotmesis 1–2 weeks after the injury but cannot distinguish neurotmesis from axonotmesis; US evaluation can make that distinction (13). Combining EDX and US can provide critical information in planning management of patients with nerve injuries. Prompt identification of nerve laceration is crucial as it requires surgical repair, while there is controversy about treatment for nerves with neuroma in continuity (7). A total of 70% of nerves in continuity regain function after 3–9 months of observation according to Pannell et al. (7).

We report 22 patients with GSWs of the upper extremities leading to nerve injuries and were referred for EDX studies. Sixteen (73%) patients also had US studies. The presenting symptoms, clinical and EDX findings, and US features of these patients are highlighted. The value of EDX and US studies in the evaluation of firearm wounds of the upper extremities as well as management of these type of injuries are discussed. We also describe the impact of the COVID-19 pandemic on the increased incidence of firearm injuries both nationally and among patients seen in our facility.

## Methods

### Study population and electrodiagnostic/ultrasound studies

This was a retrospective study under an Institutional Review Board (IRB)-approved protocol. Inclusion criteria included patients referred to our facility for EDX studies to evaluate for the presence of a nerve injury after sustaining a GSW to the upper extremities during the 8-year (2016–2023) period. The patients underwent clinical neurological examination followed by nerve conduction and EMG studies. The EDX studies were performed in our American Association of Neuromuscular and Electrodiagnostic Medicine (AANEM)-accredited facility using standard protocol of our laboratory (14). Patients in whom EDX studies did not show evidence for nerve injuries were excluded from the study. The US studies were conducted using the GE Logiq E system and 12–18 MHz probe (six patients were seen before the US machine was available to us). Short axis views at, proximal to, and distal to the injury were obtained to evaluate the cross-sectional area (CSA), fascicular pattern, and altered echogenicity (15). Long axis views were also studied. Several metrics were collected including the patients' gender and age, location of projectile entry, the specific nerve(s) injured, and the type of injury (neuropraxia, axonotmesis, or neurotmesis).

### Ethical approval and informed consent

Informed consent was obtained from all patients. The IRB determined that our study was exempt according to 45 CFR 46.101(b) under Category 4. The IRB number is 22.1087.

## Results

### Clinical findings

**Figure 1:**
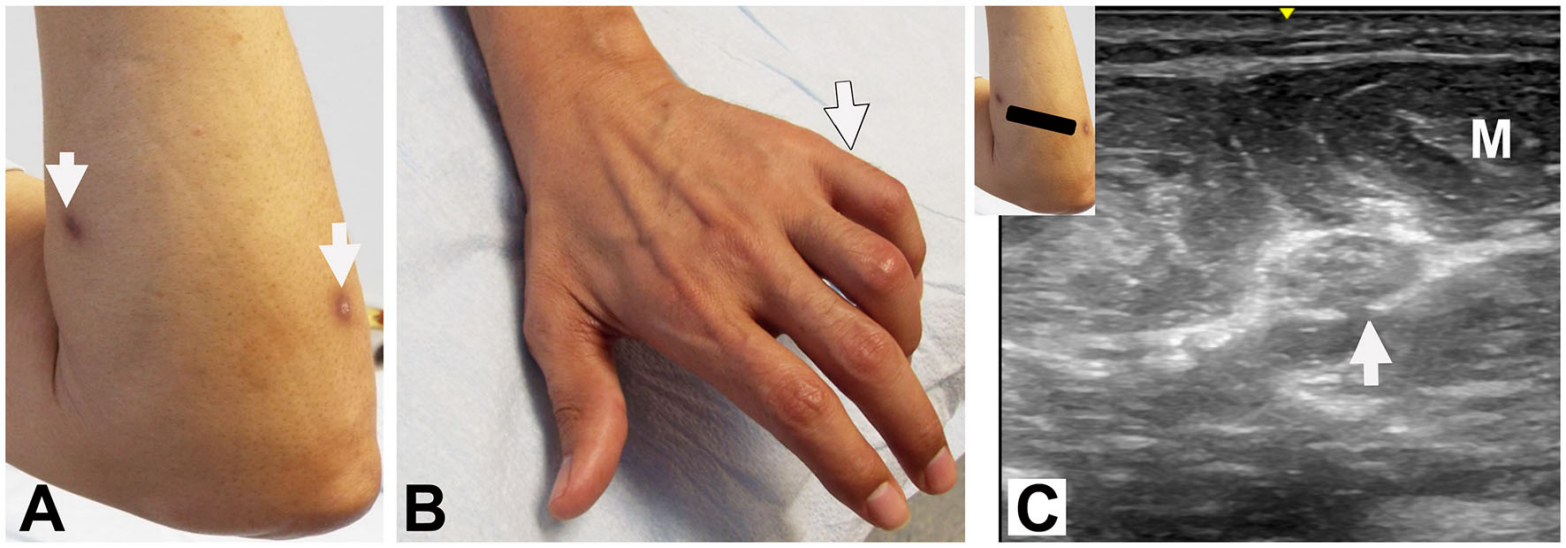
Patient #4: **(A)** Entry: dorsal proximal forearm (right arrow). Exit: ventral proximal forearm (left arrow). **(B)** Clawing of digits 4 and 5 (arrow). **(C)** Ultrasound: Short axis view at proximal forearm shows enlarged ulnar nerve (cross-sectional area 18mm^2^) (arrow) with loss of fascicular pattern. M: Flexor carpi ulnaris muscle.

**Figure 2:**
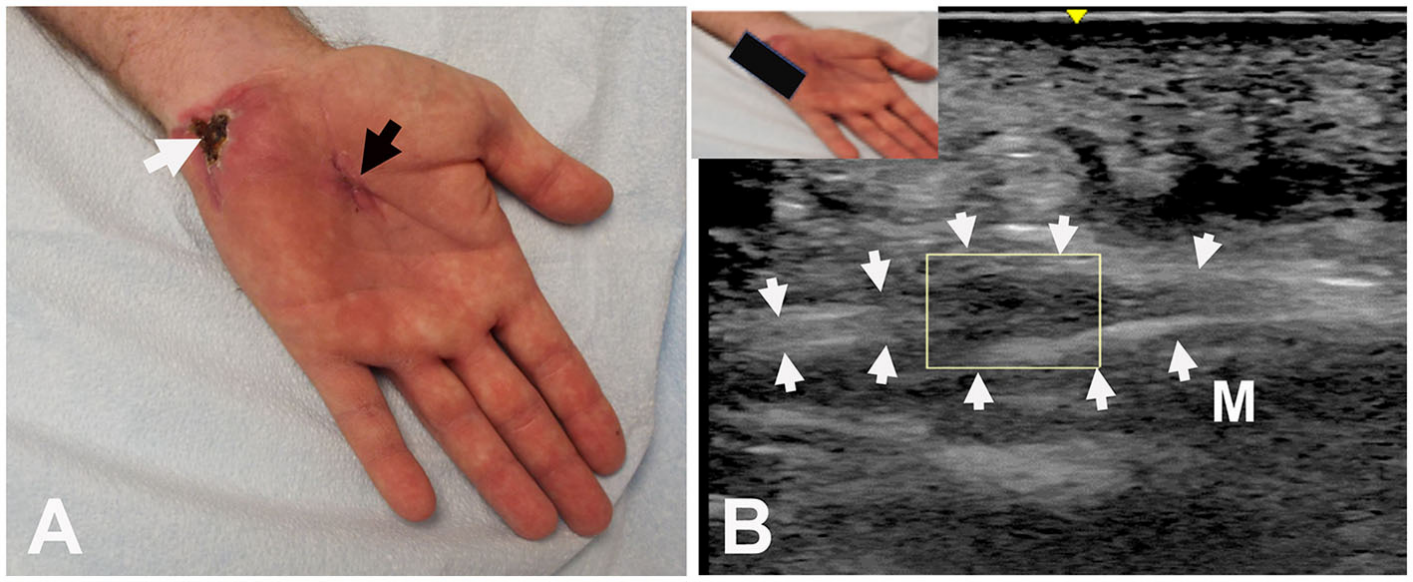
Patient #9: **(A)** Entry in palm (black arrow) and exit at base of hypothenar eminence (white arrow). **(B)** Ultrasound: Long axis view shows hypoechoic enlargement of the ulnar nerve (neuroma in continuity) (box). Arrows outline the ulnar nerve. M: Abductor digiti minimi muscle.

A total of 22 patients sustained gunshot injuries of the upper extremities (Table 2). The mean age was 38 years (range: 14–68 years), and 19 (86%) patients were male. The left side was more commonly (13 [59%] patients) injured. A total of 21 (95%) patients had single projectile entry, while one (5%) had three entries of the same upper extremity. Another six (73%) patients sustained both the entry and exit wounds in the same upper extremity (Figures 1A–C, 2A). The forearm was the most frequent entry wound location (8 [36%] patients). The ulnar nerve was the most commonly injured nerve (10 [45%] patients), followed by the brachial plexus (seven [32%] patients). Nine (41%) patients suffered GSWs of the upper extremities prior to the COVID-19 pandemic, while there were 13 (59%) victims after the COVID-19 onset.

**Table 2:** Demographics of patients with gunshot-related nerve injuries to the upper extremities referred for EDX studies.

**Patient #**	**Year**	**Gender**	**Age (years)**	**R/L**	**Location of projectile entry**	**Nerve(s) injured**
1	2016	M	29	L	Forearm	Ulnar
2	2016	M	52	L	Forearm	PIN
3	2016	M	57	L	Forearm	Radial
4	2017	M	63	L	Forearm	Ulnar
5	2018	F	38	L	Infraclavicular	Brachial plexus
6	2018	M	68	L	Forearm	Median, PIN
7	2019	M	37	L	Supraclavicular	Brachial plexus
8	2019	F	31	L	Clavicle	Brachial plexus
9	2019	M	47	L	Ulnar palm	Ulnar (Guyon)
10	2020	M	27	R	Forearm, hand, upper arm	Ulnar, Radial, Median
11	2020	M	19	R	Upper arm	Ulnar, Radial, Median
12	2021	F	14	L	Forearm	Ulnar
13	2021	M	33	R	Radial wrist (entry), hypothenar (exit)	Ulnar, Median
14	2021	M	34	R	Supraclavicular (entry); infrascapular (exit)	Brachial plexus
15	2021	M	38	R	Upper arm (entry), right side of neck (exit)	Brachial plexus
16	2021	M	24	R	Elbow	Ulnar, Median, Medial antebrachial cutaneous
17	2021	M	50	L	Proximal forearm, traversed elbow, lodged over deltoid	Radial
18	2021	M	56	L	Palm of hand (entry), dorsal hand (exit)	Ulnar (deep branch), Median digital branches
19	2022	M	48	L	Infraclavicular	Brachial plexus (Posterior cord, Suprascapular)
20	2023	M	24	R	Elbow (entrance), forearm (exit)	Ulnar, PIN
21	2023	M	15	R	Upper arm	Median
22	2023	M	33	R	Scapular area (entrance), deltopectoral groove (exit)	Brachial plexus

R, Right; L, Left; GSW, Gunshot Wound; PIN, Posterior Interosseous Nerve.

**Figure 3:**
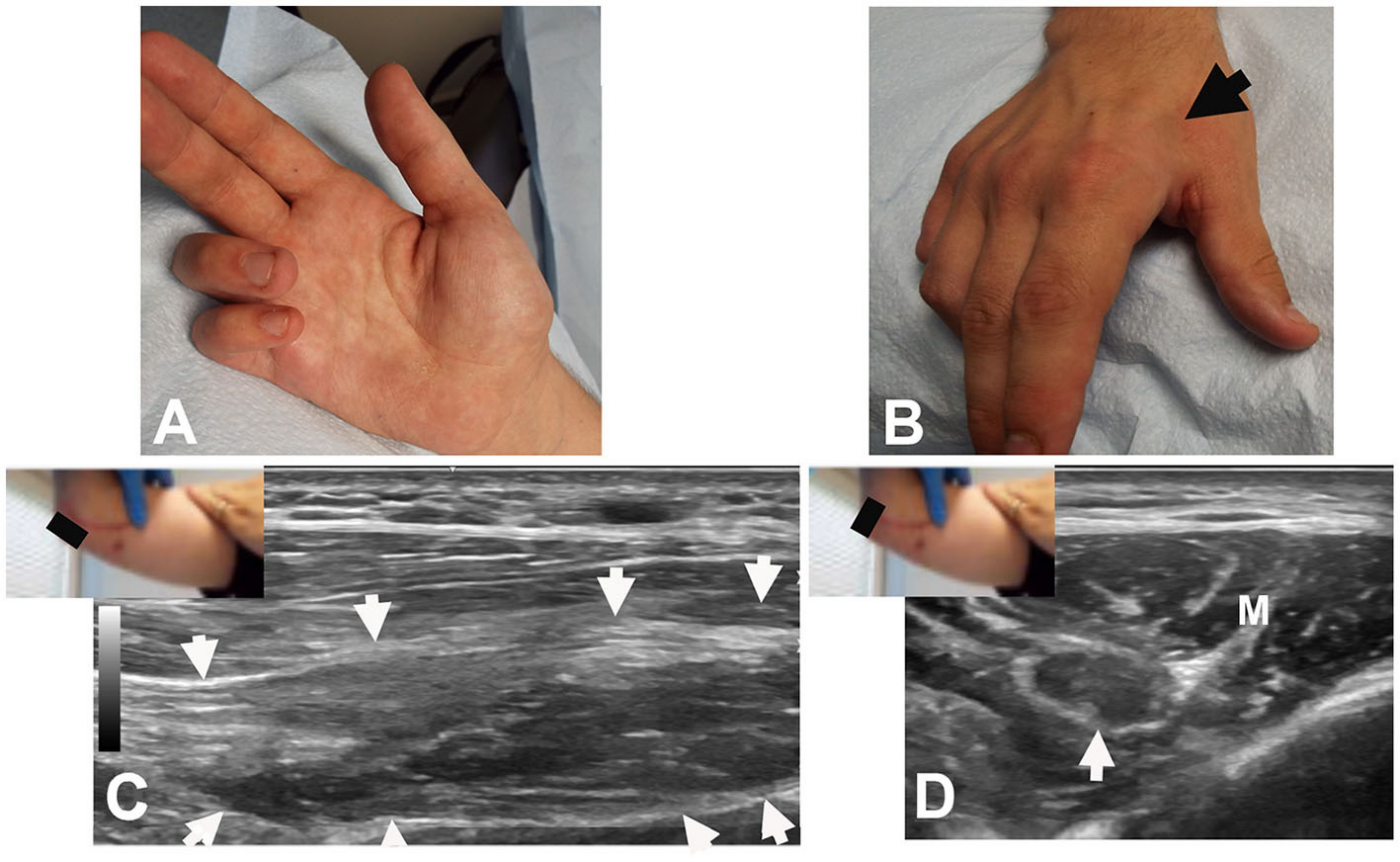
Patient #20: **(A)** Clawing of ulnar 2 digits of right hand. **(B)** Atrophy of FDI (arrow). **(C)** Long axis view at the elbow showing neuroma in continuity (arrows). The black rectangle denotes the position of the US probe. **(D)** Short axis view at the elbow showing the neuroma (arrow); the nerve is hypoechoic with epineural thickening. Note the transition from fascicular edema (on the left) to irregular echotexture (to the right). M: Flexor carpi ulnaris muscle.

All patients experienced significant muscle weakness of the upper extremities following the gunshot injuries (Table 3). The most frequently affected muscles that caused decreased or absent muscle contraction were the first dorsal interosseous (FDI) (14 [64%] patients), abductor pollicis brevis (APB) (11 [50%] patients), and abductor digiti minimi (ADM) and extensor digitorum communis (EDC) (each 7 [32%] patients). Muscle atrophy (clinical observation) was noted in 19 (86%) patients, 15 of whom had atrophy of the FDI (Figures 3A–D). A total of 19 of the 22 patients showed sensory loss corresponding to the nerve injured.

**Table 3:** Clinical findings of patients with gunshot-related nerve injuries to the upper extremities.

**Patient #**	**Side**	**Muscle strength**	**Muscle atrophy**	**Decreased pinprick sensation**	**Nerve(s) injured**
1	L	0/5 FDI, ADM; 5/5 FCU	FDI	Ulnar 2 digits, ulnar palm	Ulnar
2	L	2-3/5 EDC, EPL, ECU; 5/5 ECRL, Triceps	None	None	PIN
3	L	0/5 EDC, Brachioradialis; 4/5 APB, PT; 5/5 Triceps, Biceps, FDI	None	None	Radial
4		0/5 ADM, FDI; 5/5 FCU	FDI	Ulnar 2 digits, ulnar palm	Ulnar
5	L	0/5 FDI, APB, EI, EDC, FCR, FCU, EI, 4/5 Triceps, Biceps, Deltoid	APB, FDI	All digits, Palm, and dorsum of hand, volar and dorsal forearm	Brachial plexus
6	L	0/5 APB; 3/5 FPL, FDP (M); 4/5 PT, EDC, ECU; 5/5 ECRL, Biceps, triceps	APB	Radial 2 digits	Median, PIN
7	L	0/5 Deltoid, biceps, infraspinatus, brachioradialis; 4/5 PT, FCR; 5/5 FDI, APB, EDC, Serratus anterior	Deltoid	Lateral forearm	Brachial plexus
8	L	0/5 Deltoid, Triceps, brachioradialis, FDI; 0/3 Biceps, APB, EDC	Deltoid, Biceps, APB, FDI	Lateral forearm	Brachial plexus
9	L	0/5 ADM, FDI; 5/5 FCU, APB	FDI	Small finger, ulnar palm	Ulnar (Guyon)
10	R	0/5 APB, FDI, EI; 3/5 EDC, FDP	APB, FDI	All digits	Ulnar, Radial, Median
11	R	0/5 FDI, APB, EI; 3/5 FPL, EDC, brachioradialis	FDI, APB	Medial forearm	Ulnar, Radial, Median
12	L	0/5 ADM, FDI; 5/5 FCU, APB, EDC	FDI	Ulnar 2 digits, ulnar palm	Ulnar
13	L	0/5 APB, FDI, ADM; 5/5 FPL, PT, FDP, EDC	APB, FDI, ADM	All digits	Ulnar, Median
14	R	2/5 APB, FDI, EDC, Biceps, Triceps, Deltoid	APB, FDI	Medial forearm	Brachial plexus
15	R	0/5 APB, FDI, EDC; 3/5 Deltoid, Triceps; 4/5 Biceps, Infraspinatus	FDI, APB	Medial forearm	Brachial plexus
16	R	0/5 APB, ADM, FDI; 2/5 PT, FDP, FCU; 5/5 EI, EDC	APB, FDI	All digits, palm, medial forearm	Ulnar, Median, Medial antebrachial cutaneous
17	L	0/5 EDC, EI, ECRL; 3/5 Brachioradialis; 5/5 Triceps, Biceps, APB, FDI	None	Radial 2 digits, anatomical snuff box	Radial
18	L	0/5 FDI; 5/5 ADM, APB	FDI	Digits 2,3,4	Ulnar (deep branch), Median digital branches
19	L	0/5 Deltoid; 4/5 infraspinatus; 4/5 Triceps; 5/5 EDC, APB, FDI, Biceps	Deltoid	None	Brachial plexus (Posterior cord, Suprascapular)
20	R	0/5 FDI, ADM, FCU; 4/5: EDC; 5/5 APB, Brachioradialis	FDI, ADM	Ulnar 2 digits, ulnar palm	Ulnar, PIN
21	R	0/5 APB, FPL; 3/5 PT	APB	Radial 4 digits, radial palm	Median
22	R	0/5 APB, FDI, EDC, Biceps, Triceps, Deltoid, Pectoralis major, Lat dorsi; 5/5 Trapezius	APB, FDI	All digits, palm, dorsum, volar forearm, upper arm	Brachial plexus

FDI, First Dorsal Interosseous; ADM, Abductor Digiti Minimi; FCU, Flexor Carpi Ulnaris; EDC, Extensor Digitorum Communis; EPL, Extensor Pollicis Longus; ECU, Extensor Carpi Ulnaris; ECRL, Extensor Carpi Radialis Longus; APB, Abductor Pollicis Brevis; PT, Pronator Teres; EI, Extensor Indicis; FCR, Flexor Carpi Radialis; FPL, Flexor Pollicis Longus; FDP, Flexor Digitorum Profundus.

### Electrodiagnostic studies

Denervation of muscles as well as absence/decreased amplitude of compound muscle action potential (CMAP) and sensory nerve action potential (SNAP) suggested axonal injury in all patients. Distinction between axonotmesis and neurotmesis could not be made based on the EDX findings.

### Ultrasound studies

**Figure 4:**
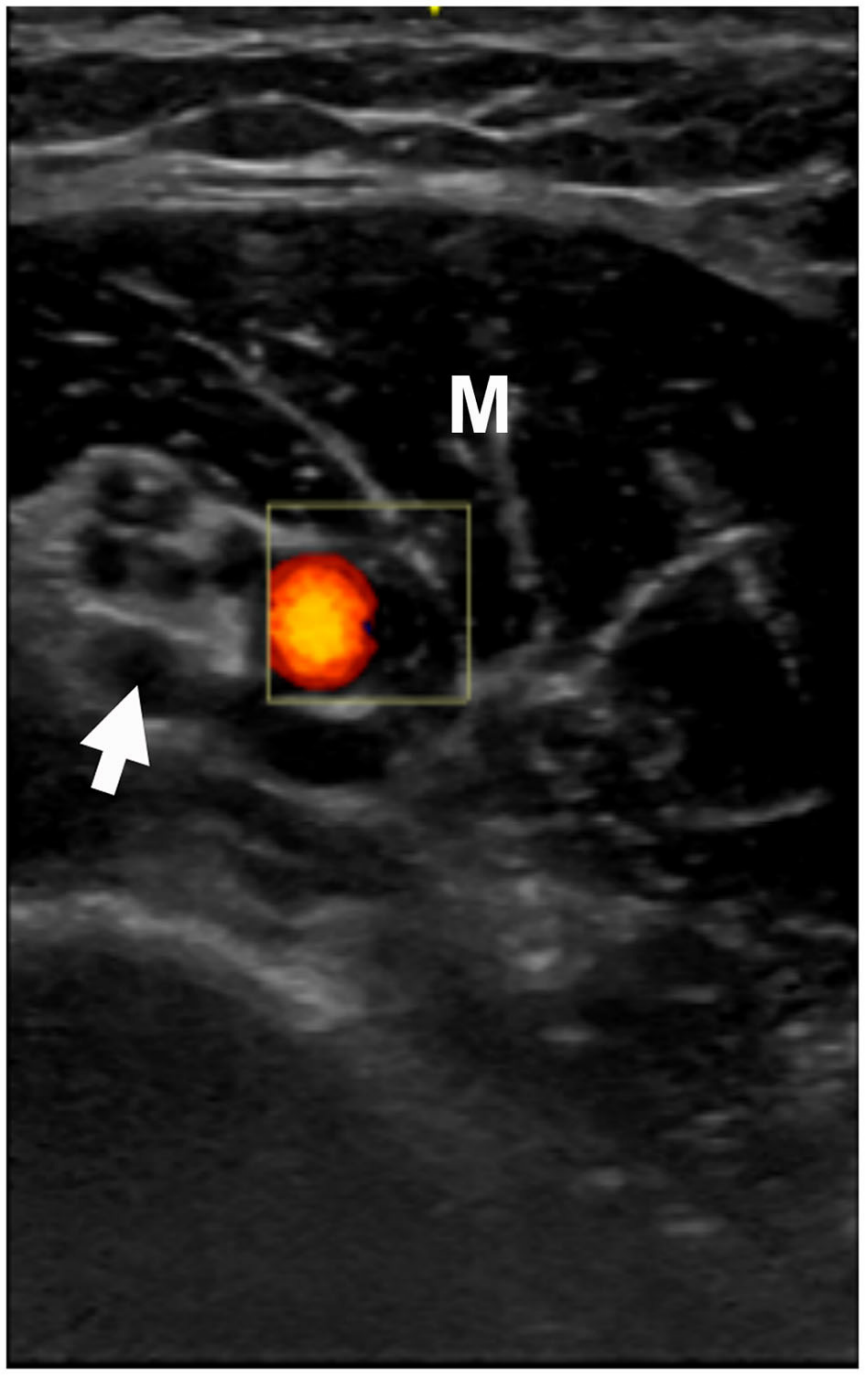
Patient #21: Ultrasound: short axis view at distal upper arm. Arrow points to the median nerve with hypoechoic enlarged fascicles and perineural thickening. The brachial artery is in orange color. M: Biceps muscle.

**Figure 5:**
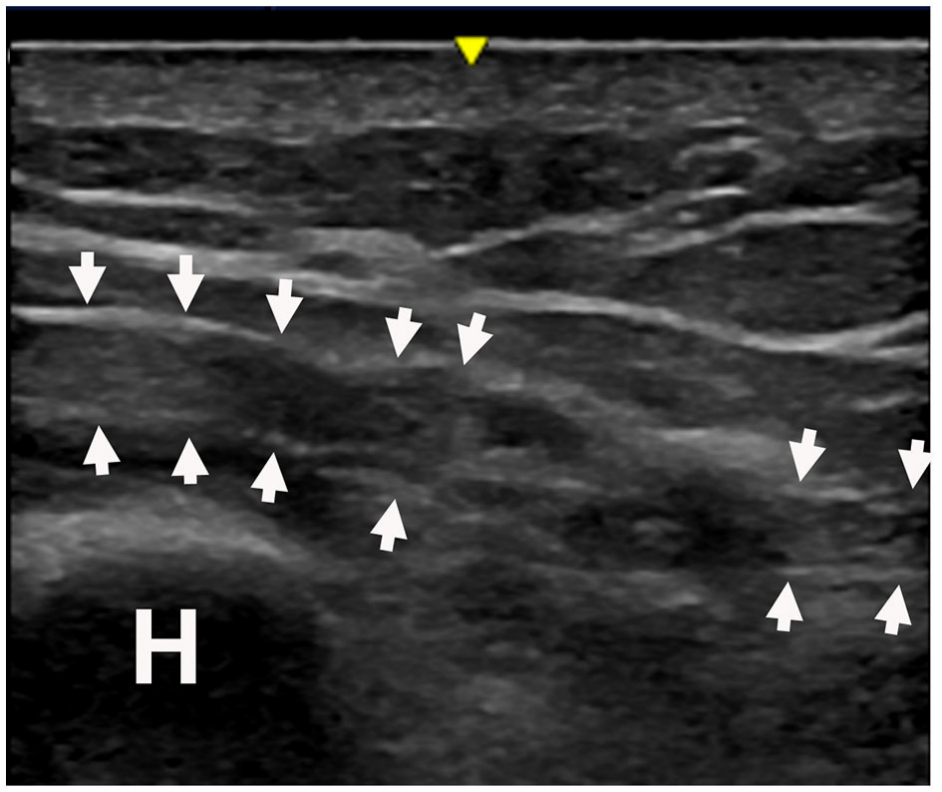
Patient #17 sustained a radial nerve injury (neurotmesis) at the upper arm. Figure shows terminal neuromas in the proximal and the distal portions of the injured nerve. H, Humerus.

Of the 16 patients who underwent US studies, a neuroma in continuity (Figures 2B, 3C) was observed in four (25%) patients (Table 4). Eleven (69%) patients had enlarged and/or hypoechoic nerves, often with large fascicles (Figure 4). One patient showed features of neurotmesis (Figure 5). Other findings included epineural and perineural thickening (hyperechoic appearance), loss of fascicular pattern and fascicular discontinuity (Table 4).

**Table 4:** Neurological and ultrasound findings of patients with gunshot-related nerve injuries of the upper extremities.

**Patient #**	**Side**	**Nerve(s) injured**	**EDX features**	**Ultrasound findings**
1	L	Ulnar at forearm	Axonotmesis	1b, 2,3,4,7
2	L	PIN at dorsolateral forearm	Axonotmesis	2,4,7
3	L	Radial at elbow	Axonotmesis	ND
4	L	Ulnar at proximal forearm	Axonotmesis	1b,2,3,4,7
5	L	Brachial plexus (predominantly lower trunk)	Axonotmesis	1b, 3,4,5
6	L	Median, PIN at forearm	Axonotmesis (Median)	2, 6
7	L	Brachial plexus (predominantly upper trunk)	Axonotmesis (mostly upper trunk)	4,5,6
8	L	Brachial plexus (predominantly posterior cord and partly medial cord)	Axonotmesis	6
9	L	Ulnar at palm (Guyon)	Axonotmesis	1a,6
10	R	Ulnar, Radial, Median at multiple sites	Axonotmesis	2,4,5,6
11	R	Ulnar, Radial, Median at upper arm	Axonotmesis	ND
12	L	Ulnar at midforearm	Axonotmesis	1b
13	L	Ulnar, Median at wrist	Axonotmesis	1a,2,3,4,5,7
14	R	Brachial plexus (pan plexus)	Axonotmesis	ND
15	R	Brachial plexus (predominantly lower trunk)	Axonotmesis	ND
16	R	Ulnar, Median, Medial antebrachial cutaneous at elbow	Axonotmesis	1a,2,3,4,5,7
17	L	Radial at upper arm	Axonotmesis	8
18	L	Ulnar (deep branch), Median digital branches at palm	Axonotmesis	ND
19	L	Brachial plexus (posterior cord, suprascapular)	Axonotmesis	ND
20	R	Ulnar, PIN at elbow, forearm	Axonotmesis	1b
21	R	Median at upper arm	Axonotmesis	2,6
22	R	Brachial plexus (pan plexus)	Axonotmesis	6

ND, Not done; L, Left; R, Right; PIN, Posterior Interosseous Nerve; 1a, neuroma in continuity with fascicular continuity; 1b, neuroma in continuity without fascicular continuity; 2, increase in cross-sectional area; 3, epineural thickening, hyperechoic; 4, interfascicular hyperechoic tissue; 5, perineural thickening, hyperechoic; 6, large fascicules, hypoechoic; 7, loss of fascicles, hyperechoic; 8, neurotmesis.

## Discussion

### COVID-19 and GSWs

According to the Morbidity and Mortality Weekly Report, the firearm homicide rate in the United States increased by approximately 35% during the COVID-19 pandemic (16, 17). Compared with 2019, the average number of weekly Emergency Department (ED) visits for firearm injuries was 27% higher in 2020, 36% higher in 2021, and 20% higher in 2022 based on data from the National Vital Statistics System (17). The highest rate was observed among individuals ages 15–24 years. Similarly, we observed a higher number of upper extremities GSW injuries following the COVID-19 initiation compared to before COVID-19 (13 vs. nine patients, respectively), with eight patients evaluated in 2021 alone. This finding of the largest number of cases in 2021 concurs with the substantial increase in ED visits for firearm injuries specifically in 2021 as reported by the National Vital Statistics System. In Risinger and colleagues' study (2011–2021) of the association between COVID-19 and gun violence in our same county, the years of potential life lost was higher for firearm fatalities than COVID-19 (3). These authors attributed the increase in gun violence during COVID-19 primarily due to stay-at-home orders and decreased access to mental health care. Additional psychodynamic factors include diminished security and safety (housing and financial insecurity, elevated exposure to violence, fear of illness, uncertainty about the future), and increased firearm purchases (17). Numerous measures have been proposed to address the risk of gunshot injuries, including enhancing community and street outreach programs, initiating implement hospital-based violence prevention programs, enriching community physical environments, encouraging secure storage of firearms, and boosting social and economic supports (17).

### Common nerves injured

Previous studies have reported gunshot injuries of the upper extremities with consequent peripheral nerve injuries, specifically, brachial plexus (6, 18, 19), radial nerve (5, 10, 20), ulnar nerve (12, 21), or a combination of upper extremity nerves (7, 8, 11, 22–25). In Secer and colleagues' 40-year study of 2,106 patients with 2,210 peripheral nerve lesions of the upper and lower extremities caused by combat-related GSWs, the median nerve was the most frequently injured upper extremity nerve followed by the ulnar nerve, radial nerve, and brachial plexus (8). In Pannell and colleagues' study (2007–2014) of 41 patients (59 nerves) who sustained firearm injuries of the upper extremities, there was a 63% incidence of nerve dysfunction after upper extremity GSWs (7). A higher frequency of fractures, retained fragments, vascular injury, and compartment syndrome in patients with nerve palsies was noted (7). Additionally, patients with palsies were significantly more likely to have nerve lacerations intraoperatively. Of the 59 nerves studied, 37 palsies were identified, including 13 ulnar, 8 median, and 7 radial. Ten of the 37 (27%) palsies had lacerated nerves, whereas nerve lacerations were not observed in patients without palsies. Straszewski et al. studied GSWs of the upper extremities in an urban trauma center; among 126 injuries in 117 patients, 38 had a documented nerve deficit (9). The location of injury was 18 in the arm, 13 in the forearm, six in both the arm and forearm, and one in the hand. The most common nerve injured was the radial nerve, followed by the ulnar and median nerves. In patients with a GSW to the forearm, the most common nerve injured was the ulnar nerve. In our study the most frequent nerve injured was the ulnar nerve, and the site of projectile entry was three at the forearm, two at the elbow, two at the palm, one at the wrist, and one at the upper arm. Henriques et al.'s study also noted the high frequency of ulnar nerve involvement (21). It is possible that the superficial location of the nerve at the elbow and the forearm makes it more vulnerable to injury.

The brachial plexus was the second most common site of GSW in our series. The projectile entry location included supraclavicular, clavicular, and infraclavicular areas; in one patient the projectile entered in the upper arm and exited on the left side of neck. Most brachial plexus injuries are due to traction injuries caused by motor vehicle accidents, however, ~3–12% result from GSWs (6, 19).

The radial/PIN nerve was injured in six patients in our study; in three patients, additional nerves were injured. In Guo et al.'s study of patients who sustained a gunshot injury of the radial nerve, only 30% of firearm radial nerve injuries were associated with another nerve injury in the upper extremity (5).

### EDX studies

EDX studies play a valuable role in the investigation of gunshot injuries by detecting nerve injuries and recovery. They localize the site of the lesion, identify the type and severity of the lesion, and provide prognostic details pre- and postoperatively (6, 19, 26). EDX studies are crucial in differentiating conduction bock from axonal injury; they are also useful in differentiating total from partial injuries and to detect reinnervation. Serial EMGs are beneficial in analyzing the progression of recovery by assessing the presence of volitionally-recruited motor units and the quantity of fibrillation potentials (19). An important limitation of EDX is its inability to distinguish total axonotmesis from neurotmesis; combining EDX and US studies can potentially circumvent this limitation and lead to prompt surgical repair without delay in cases of neurotmesis.

In this series, the findings suggested axonotmesis in all patients. The EDX studies were performed in most patients 3 months after injury (ranging from 2 months to 12 months). Injuries of the brachial plexus were partial and involved trunks/cords.

### Ultrasound study

An MRI is the gold standard for imaging nerve injuries of the upper extremities due to its multiplanar images and high-contrast resolution in soft tissues (6). However, this modality may be contraindicated in GSWs due to the presence of metal fragments which may shift, generate heat, and may cause imaging artifact. The readily available and cost-effective US may be a more useful technique to detail the anatomy of the injury without being limited by metal fragments. US also detects both nerve and vascular injury as well as the presence of neuroma formation and nerve structural integrity following firearm injuries (6, 27). In Fagan and colleagues' study of 17 patients who sustained a GSW of the upper or lower extremities and subsequently underwent neuromuscular US, all patients had either a nerve transection or neuroma in continuity of a major upper or lower extremity nerve at the site of the GSW (27). A total of 13 (76%) patients had significant morphological changes in distal segments of the injured nerve, and 12 (71%) had changes in other nearby distal nerves. Enlarged nerve cross-sectional area, enlarged fascicles, and hypoechogenicity were also frequently observed. These authors surmised that ballistic trauma of firearm injuries can result in concussive damage that disrupts the normal architecture of distal nerves (27). In Nwawka and colleagues' case series of 3 patients who sustained brachial plexus injuries due to firearms, the US revealed nerve abnormalities that complemented the EDX and intraoperative findings (6). Two of these patients experienced nerve transection of the median and ulnar nerves detected by US, one of whom also had a radial nerve transection. In this series four patients with brachial plexus injury showed hypoechoic enlarged trunks, perineural thickening and hyperechoic scar tissue surrounding the neural structures. Of the 16 patients who underwent US in the present study, a neuroma in continuity was observed in four patients, while 11 patients had enlarged and/or hypoechoic nerves. In one patient neurotmesis was noted (Table 4).

Among the advantages of US in comparison to MRI, dynamic evaluation can be useful for confirming neurotmesis and monitoring the recovery process. Additionally, US may detect compression by a hematoma and entrapment by scar tissue formation (28, 29).

### Strengths and limitations

The strength of our study is the use of both EDX and US studies in evaluating a large cohort of gunshot-related nerve injuries of the upper extremities in a non-combat setting. This allowed us to correlate the clinical examination findings with the EDX and US features in these patients. Limitations of our study include its retrospective nature and lack of follow-up of patients after their EDX evaluation which has precluded our ability to assess the long-term outcome. Additional limitations include the lack of measuring the cross-sectional area of the different nerves using comparative scanning, the lack of color/power Doppler assessment of the pathological peripheral nerves, and the lack of sonographic follow-up.

## Conclusion

Physicians should be cognizant of the potential for nerve injuries following GSWs and look for the clinical signs, diagnostic of injury to specific nerves. EDX and US studies should be utilized to determine the location and the type of nerve injury so that appropriate management can be initiated promptly.

## Data availability statement

The original contributions presented in the study are included in the article/supplementary material, further inquiries can be directed to the corresponding author.

## Ethics statement

The studies involving humans were approved by University of Louisville Institutional Review Board. The studies were conducted in accordance with the local legislation and institutional requirements. The participants provided their written informed consent to participate in this study.

## Author contributions

LS: Conceptualization, Data curation, Formal analysis, Investigation, Methodology, Project administration, Resources, Software, Supervision, Validation, Visualization, Writing – original draft, Writing – review and editing. VI: Conceptualization, Data curation, Formal analysis, Investigation, Methodology, Project administration, Resources, Software, Supervision, Validation, Visualization, Writing – review and editing. YZ: Resources, Software, Visualization, Writing – review and editing. CS: Conceptualization, Data curation, Formal analysis, Investigation, Methodology, Project administration, Resources, Software, Supervision, Validation, Visualization, Writing – review and editing.
